# Bullose diabétique

**DOI:** 10.11604/pamj.2016.23.15.8604

**Published:** 2016-01-26

**Authors:** Najia Ilham El Makrini, Badredine Hassam

**Affiliations:** 1 Service de Dermatologie et de Vénéréologie, Centre Hospitalier Ibn Sina, Rabat, Maroc

**Keywords:** Diabète, bulleuse, neuropathie

## Image en medicine

La bullose des diabétiques fait partie des complications cutanées du diabète, c'est une dermatose bulleuse rare, particulière par sa survenue exclusive chez le diabétique. Nous rapportons le cas d'une patiente âgée de 59 ans ayant comme antécédent un diabète sous insuline depuis 30 ans et une pleurésie pulmonaire traitée il y a 3 ans et qui consultait pour des lésions bulleuses d'apparition récente et spontanée, localisées au niveau des mains (A) et des pieds (B), à contenu clair ou hémorragique. Cliniquement, il existait aussi une neuropathie périphérique. L’évolution sous traitement symptomatique était favorable. La bullose diabétique est une pathologie rare qui survient chez 0,5 % des diabétiques et pourrait s'expliquer par les altérations vasculaires qui seraient à l'origine d'une fragilité cutanée ainsi du clivage de la peau. La présence fréquente d'une neuropathie périphérique pourrait aussi expliquer la localisation préférentielle distale des lésions. Sur le plan thérapeutique, aucune conduite particulière ne semble nécessaire en dehors des soins locaux antiseptiques après l’évidement des bulles. La bullose des diabétiques est une complication cutanée caractéristique du diabète. Son étiologie est encore inconnue. Son évolution est bénigne et sa prise en charge reste essentiellement préventive.

**Figure 1:**
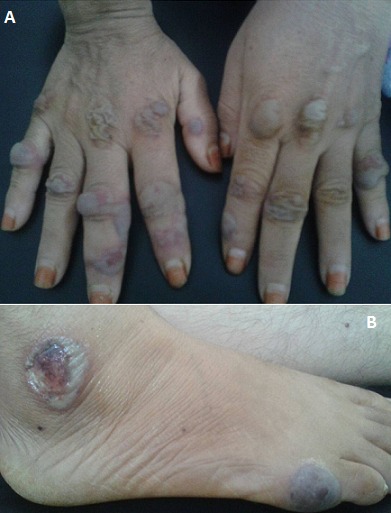
A) lésions bulleuses des mains; B) lésions bulleuses en regard des chevilles

